# Kartogenin hydrolysis product 4-aminobiphenyl distributes to cartilage and mediates cartilage regeneration

**DOI:** 10.7150/thno.38182

**Published:** 2019-09-21

**Authors:** Shuai Zhang, Peilin Hu, Tao Liu, Zhen Li, Yongcan Huang, Jinqi Liao, Md Rana Hamid, Liru Wen, Ting Wang, Cuiping Mo, Mauro Alini, Sibylle Grad, Tianfu Wang, Di Chen, Guangqian Zhou

**Affiliations:** 1 Department of Medical Cell Biology and Genetics, Guangdong Key Laboratory of Genomic Stability and Disease Prevention, Shenzhen Key Laboratory of Anti-aging and Regenerative Medicine, and Shenzhen Engineering Laboratory of Regenerative Technologies for Orthopaedic Diseases, Health Sciences Center, Shenzhen University, Shenzhen, 518060, China.; 2 Department of Biotherapy and Oncology, Shenzhen Luohu People's Hospital, Shenzhen, 518001, China.; 3 AO Research Institute Davos, Davos, Switzerland.; 4 Shenzhen Engineering Laboratory of Orthopaedic Regenerative Technologies, Departments of Orthopaedics, Peking University Shenzhen Hospital, Shenzhen, Guangdong, 510086, China.; 5 Shenzhen Apls Cell Technologies LTD., Yinxing Scientific Building, Lonhua District, Shenzhen, 510086, China.; 6 Guangdong Key Laboratory for Biomedical Measurements & Ultrasound Imaging, School of Biomedical Engineering, Health Sciences Center, Shenzhen University, Shenzhen, 518060, China.; 7 Department of Orthopedic Surgery, Rush University Medical Center, Chicago, IL 60612, USA.

**Keywords:** kartogenin, 4-aminobiphenyl, stem cells, osteoarthritis, RPS6KA2

## Abstract

**Rationale** The small molecule Kartogenin (KGN) promotes cartilage regeneration in osteoarthritis (OA) by activating stem cells differentiation, but its pharmacological mode-of-action remains unclear. KGN can be cleaved into 4-aminobiphenyl (4-ABP) and phthalic acid (PA) following enzymolysis of an amide bond. Therefore, this study investigated whether 4-ABP or PA exerted the same action as KGN.

**Methods** KGN, 4-ABP and PA were analyzed in cartilage of mice after oral, intravenous or intra-articular administration of KGN by liquid chromatography-mass spectrometry method. Their effect on proliferation and chondrogenic differentiation of mesenchymal stem cells (MSC) was evaluated *in vitro*. Furthermore, their effect on cartilage preservation was tested in mice OA model induced by destabilization of medial meniscus. OA severity was quantified using OARSI histological scoring. Transcriptional analysis was used to find the possible targets of the chemicals, which were further validated.

**Results** We demonstrated that while oral or intra-articular KGN delivery effectively ameliorated OA phenotypes in mice, only 4-ABP was detectable in cartilage. 4-ABP could induce chondrogenic differentiation and proliferation of MSC *in vitro* and promote cartilage repair in OA mouse models mainly by increasing the number of CD44^+^/CD105^+^ stem-cell and prevention of matrix loss. These effect of 4-ABP was stronger than that of KGN. Transcriptional profiling of 4-ABP-stimulated MSC suggested that *RPS6KA2* and the PI3K-Akt pathway were 4-ABP targets; 4-ABP could activate the PI3K-Akt pathway to promote MSC proliferation and repair OA injury, which was blocked in *RPS6KA2-*knockdown MSC or *RPS6KA2-*deficient mice*.*

**Conclusion** 4-ABP bio-distribution in cartilage promotes proliferation and chondrogenic differentiation of MSC, and repairs osteoarthritic lesions via PI3K-Akt pathway activation.

## Introduction

Osteoarthritis (OA) is a prevalent and crippling joint disease characterized by cartilage degeneration and loss and curative treatment options are lacking due to its complex pathogenesis 1, 2. Multiple OA treatment approaches utilizing culture-expanded stem cell-based therapies have been proposed but the optimal stem cell sources have not been identified and the long-term outcomes are elusive. In contrast, the native joint-resident stem cell-based therapy seems to be a promising alternative and new factors and compounds activating the endogenous stem cells are emerging 3. Joint-resident mesenchymal stem cells (MSC) reside in multiple niches, including the cartilage, bone marrow, synovial fluid, joint adipose tissue, periosteum and synovium 3, 4. The combination of BMP-2 and TGF-β strongly induces the chondrogenic differentiation of MSC and prevents HtrA1 expression 5. siRNAs targeting Col1a1, Htra1 or Runx2 are functionally validated for cartilage repair 5, 6. Furthermore, some biomaterials, such as the stem cell-laden supramolecular hydrogels, injectable gelatin hydrogels, could encapsulate MSC and drug (icaritin) to enhance selective MSC differentiation and cartilage regeneration 7, 8. Consequently, molecules that promote selective MSC proliferation and differentiation into chondrocytes may stimulate cartilage repair in OA.

Kartogenin (KGN) is a small molecule chemical that can induce cartilage endogenous MSC differentiation and protect articular chondrocytes *in vitro* and in multiple OA animal models 9-11. Further studies have confirmed the chondrogenetic action induced by KGN or KGN-conjugated to biomaterials, such as PEGylated polyamidoamine, polyurethane nanoparticles, chitosan and thermogel 12-16. Mechanistically, KGN binds to the intracellular molecule filamin A, disrupts its interaction with CBFβ, and induces chondrogenesis by regulating the CBFβ-RUNX1 transcriptional program 10. Other studies suggested KGN has pro-chondrogenic effects mainly by activating the JNK-RUNX1 pathway, and anti-osteogenic effects by suppressing the β-catenin-RUNX2 pathway 17. Besides inducing chondrogenic differentiation of stem cells, KGN has also been shown to enhance the proliferation of bone marrow mesenchymal stem cells (BMSC) and tendon stem/progenitor cells and promote the healing of injured tendon-bone junctions in rabbits 18, 19. In addition, KGN has been shown to promote proliferation of human adipose-derived stem cells and BMSC by activating the AMPK-SIRT1 signaling pathway 20, 21. Taken together, KGN may be a potential chemical for use as an endogenous stem cell-based therapy in OA; however, whether KGN itself can enter the cartilage after intra-articular injection or systemic administration remains to be determined.

KGN structurally contains 4-aminobiphenyl (4-ABP) and phthalic acid (PA) linked via an amide bond. Resonance stabilization contributes to chemical stability of the amide bond with a partial double-bond character of the C-N bond 22. Although the amide bond is generally stable, its hydrolysis is a ubiquitous process and is catalyzed by various enzymes, including allantoinases, alkaline ceramidases, amidases and peptidases 23, 24. Studies on other molecules, such as the novel organic nitrate RS-7897, have shown that hydrolysis of an amide bond can release more active molecules than the parent molecule 25. Furthermore, amide bonds are often incorporated into drug designs, as a linker to tether a drug to a targeting peptide 26.

Herein, we aimed to determine whether KGN served as a pro-drug that releases 4-ABP and/or PA as active chemicals. We specifically studied whether KGN was hydrolyzed at its amide bond into 4-ABP and PA upon their administration to cartilage-derived-stem/progenitor cells (CSPC), MSC or *in vivo* mouse models, and investigated the molecular mechanisms involved in modulating the action of KGN, 4-ABP and PA activity in cellular and animal OA models.

## Methods

### Chemicals

KGN, 4-ABP and PA were purchased from MedChemExpress (USA), Macklin Biochemical Co., Ltd (Shanghai, China) and Aladdin Biochemical Co., Ltd (Shanghai, China), respectively. BI-D1870 was purchased from MedChemExpress (USA). Fetal bovine serum (FBS), Dulbecco's modified Eagle's medium (DMEM), DMEM/F-12 and trypsin were purchased from Gibco (USA). Dexamethasone, insulin-transferrin-sodium selenite, ascorbate-2-phosphate and sodium pyruvate were obtained from Sigma-Aldrich (St Louis, MO, USA). TGF-β1 was obtained from PeproTech Inc. (USA).

### Animals

Male STR/Ort mice (Shanghai Research Center for Model Organisms, Shanghai, China), C57BL/6J mice (Guangdong Medical Laboratory Animal Center, Guangzhou, China) and *Rps6ka2* knockout mice congenic on a C57BL/6J background (Cyagen Biosciences, Guangzhou, China) were maintained in polypropylene cages and housed under standard conditions. RSK3 was encoded by *Rps6ka2* gene. RSK3^-/-^ mice were purchased from Cyagen Biosciences Inc. (Guangzhou, China). Constitutive RSK3 knockout mice were backcrossed to C57BL/6J mice over 10 generations.

All animal care and experimental procedures complied with the National Research Council's guide for the care and use of laboratory animals, and all procedures were approved by the Institutional Animal Care and Use Committee of Shenzhen University (Shenzhen, China).

### Surgery-induced osteoarthritis mouse model

A previously reported protocol was followed to create the destabilization of medial meniscus (DMM) model in mice 27. Briefly, male mice (body weight: 25 ± 3 g, n = 10 per group) were anesthetized with 2% isoflurane in air using an anesthesia machine (RWD Life Science Co., Ltd., Shenzhen, China). After opening the right knee joint capsule, the medial meniscotibial ligament, which anchored the medial meniscus to the tibial plateau, was cut to destabilize the joint. Cartilage injury beneath the medial meniscus was avoided. Then, the knee joint capsule was closed with a 6-0 suture. Knee joint capsule was only opened in the sham group. Thirty days after surgery, KGN (2.5 and 5 mg/kg, dissolved in saline) was orally administered to STR/Ort mice once a day for two months and then the mice were sacrificed.

To assess the protective effects of these chemicals in the OA model, DMM was created in C57BL/6J mice (n = 10 per group) and then 10 μM KGN, 4-ABP or PA formulated in saline was immediately administered by intra-articular injection (4 μL) once a week for 8 weeks, and the knee joints were collected for toluidine blue or safranin O-fast green staining. Osteoarthritis Research Society International (OARSI) scores were used for lesion assessment 28.

The surgery was performed by the same operator who was skilled at DMM operation and blinded to this study. All data analysis was performed in a double-blind manner by two investigators who was given no information about the experimental groups.

### Cell culture and treatment

#### Isolation and culture of murine CSPC

The CSPC mainly locate in the surface zone of articular cartilage and have higher affinity for fibronectin than other ligands, such as collagen types I and II, laminin and tenascin 4, 29. While the chondrocytes in the middle zone of cartilage are also involved in initial adhesion to fibronectin, but lack the ability to form colonies and cannot undergo continuous passage 29. According to previous reports and our previous methods 30, 31, we obtained CSPC from the cartilage of STR/Ort mice based on their adhesion to fibronectin and a high colony-forming efficiency. Murine knee joints (n = 10-15) were used to isolate CSPC from cartilage. CSPC were used at passage 4.

#### Isolation and culture of rat BMSC

Two male Sprague-Dawley rats (body weight, 200-240 g) were anesthetized with 10% chloral hydrate and sacrificed by cervical dislocation. Then, the animals were rinsed freely in 70% ethanol and the hind limbs were dissected from the trunk of the body. After removing the muscle and connective tissue from both the tibia and the femur, the bone marrow in the cavity was flushed with DMEM (low glucose) using 27-gauge needles. The cell suspension was filtered through a 70-μm filter mesh and the cells in complete medium (low-glucose DMEM medium with 15% FBS, 2 mM L-glutamine, 100 μg/mL penicillin, 100 μg/mL streptomycin) were seeded in 95-mm culture dishes. After incubation for 24 h at 37°C with 5% CO_2_ in a humidified chamber, the non-adherent cells were removed and the medium was replaced with fresh complete medium. Then, the medium was changed every 8 h for up to 72 h of initial culture, and then every 3-4 days until the cells reached 90% confluence. After several passages, the purified population of BMSC was evaluated for characteristic spindle-shaped morphology and further cell-surface marker expression by flow cytometry analysis.

#### Umbilical cord mesenchymal stem cells (UC-MSC)

Primary human UC-MSC were purchased from Nuwacell Ltd (Hefei, China) and were cultured in NuwacellTM-Misson Basal Medium with the supplements (Nuwacell Ltd, Hefei, China).

### Chondrogenic differentiation

BMSC and UC-MSC were seeded in 6-well plates and treated with KGN, 4-ABP or PA for 4 days or 20 days prior to analyses of chondrogenic differentiation potential.

CSPC (1 × 10^6^) from STR/Ort mice were centrifuged at 300 g at 4°C for 6 min in polypropylene tubes to form a pelleted micromass. The pellets were then cultured with various medium for different groups. The control group was cultured with DMEM medium. The vehicle group was cultured with DMEM medium supplemented with 100 nM dexamethasone, 1% insulin-transferrin-sodium selenite, 50 μM ascorbate-2-phosphate, 1 mM sodium pyruvate and 50 ng/mL TGF-β1 for chondrogenic differentiation. KGN, 4-ABP or PA (10 μM) was added to the chondrogenic differentiation medium to observe the effects on chondrogenic induction. After 21 days of induction, the pellets were collected for detecting chondrogenic gene expression, pellet diameters and type II collagen expression.

### High performance liquid chromatography-mass spectrometry (HPLC-MS)

High chemical dose exposure facilitates to detect the chemicals *in vivo*. So we gave the maximum effective dosage of KGN based on the current study (oral administration: 5 mg/kg) and previous report (intra-articular injection: 100 μM 10) to mice. KGN at the dose of 2.5 mg/kg (half of the oral dosage 32) was intravenously injected via tail vein. KGN was given to C57BL/6J mice (n = 3 per group) by the different administration routes for 3 days (once a day), and blood and cartilage were collected 2 h after the last dose. For blood, the samples were immediately frozen in liquid nitrogen and lyophilized with a freeze drier. Then, the powder of supernatants or cells were dissolved in 1 mL acetonitrile and then centrifuged at 8000 g for 10 min and the supernatants were used for chemical detection by HPLC-MS. Murine knee joints were dissected and the cartilage on the surface of femur and tibia was carefully excised using a scalpel under a stereoscopic microscope (Ruihoge, China) to avoid the contamination of ligament, subchondral bone and meniscus. Then the collected cartilage was crushed by trituration in liquid nitrogen, dissolved in 0.5 mL acetonitrile and then centrifuged at 8000 g for 10 min. The supernatants were used for chemical detection by HPLC-MS.

UC-MSC were seeded in 6-well plates (1 x 10^5^ cells per well) and incubated at 37°C with 5% CO_2_. After exposure to KGN (10 μM) for 24 h, the supernatants and cells were collected separately, immediately frozen in liquid nitrogen and lyophilized with a freeze drier. Then, the powder of supernatants or cells were dissolved in 1mL acetonitrile, centrifuged at 8000 g for 10 min and the supernatants were used for chemical detection by HPLC-MS.

KGN, 4-ABP and PA levels were analyzed on an LCMS 2020 system (Shimadzu Corporation, Kyoto, Japan) equipped with an electro spray ionization (ESI) source. Analyses were performed with an ACQUITY-UPLC-BEH C-18 column (Waters, 1.7 μm particle size, 100 × 2.1 mm); column temperature, 40°C; mobile phases, ammonium acetate : acetonitrile = 15 : 85; flow rate, 0.2 mL/min and injection volume, 10 μL. Mass analysis was performed using an ESI source in positive ion mode. The following conditions were used: collision gas, 1.5 L/min; dry gas, 10 L/min; detection voltage, 1.1 kV; DL temperature, 250°C; ion source temperature, 350°C; and heat block temperature, 450°C. The chemicals were identified on the basis of the retention times and mass spectra of the chemical standards. To improve the sensitivity for chemicals, the selected ion monitoring mode was used. The characteristic ions (KGN, m/z = 299; 4-ABP, m/z = 169; PA, m/z = 104) were selected for quantitation analysis.

### Cell proliferation

Cell viability was analyzed using a Cell Counting Kit-8 (CCK-8) (MedChemExpress, USA). Briefly, 3,000 cells were seeded in 96-well plates. After culture for 0, 1, 2 and 3 days, CCK-8 solution was added to each well and incubated at 37°C for 1 h. The optical density at 450 nm was determined using a microplate reader (Multiskan GO, Thermo Scientific, Germany).

### Flow cytometry

To confirm UC-MSC and rat BMSC characteristics, positive (CD73, CD105, CD44 and CD90) and negative (CD79α, HLA-DR, CD14, CD45, CD34) cell-surface markers were detected by flow cytometry. The cells (1 × 10^6^) were harvested by trypsin digestion, washed with phosphate buffer saline (PBS), blocked with 2% FBS at 4°C for 30 min and then incubated individually with FITC/PE/APC-conjugated antibodies (BD Biosciences, USA) at 4°C for 1 h. Unlabeled cells were used as a control for all antibodies. The cells were re-suspended in 0.4 mL PBS and analyzed on a FACSCalibur (Becton Dickinson, USA). Data were processed using FlowJo software (Java Software).

### RNA-sequencing and bioinformatics analysis

UC-MSC (1 x 10^5^ cells per well) were seeded in 6-well plates, grown to 80% confluence and treated with KGN or 4-ABP (10 μM) in DMEM/F-12 and 15% FBS medium for 3 days. Then, total RNA from cells of each group was extracted using TRIzol reagent (Invitrogen), according to the manufacturer's instructions. RNA-sequencing and bioinformatics analyses were performed as previously described 30.

### shRNA-mediated gene knockdown

Mouse *RPS6KA2* gene short hairpin RNA (shRNA) sequences were designed according to the cDNA sequence using the online software (http://bioinfo.clontech.com/rnaidesigner/frontpage.jsp). After multiple testing and verification, one pair of designed shRNA (*RPS6KA2*-S2: GATCCGAGATAGACATCAGCCATCTTCAAGAGAGATGGCTGATGTCTATCTCTTTTTT; *RPS6KA2*-A2: AATTAAAAAAGAGATAGACATCAGCCATCTCTCTTGAAGATGGCTGATGTCTATCTCG) was chosen for this study. After synthesis and annealing, four double-stranded oligonucleotides (dsOligo) were cloned into the pDC316-gfp-U6 plasmid (Miaoling Bioscience & Technology, Wuhan, China), and the sequences were confirmed by PCR and DNA sequencing. Real-time PCR and Western-blotting were used to screen the most effective pDC316-gfp-*RPS6KA2*-shRNA plasmid in HEK293 cells, and the most effective plasmid was packaged into the recombinant adenovirus AD-RSK3-shRNA with adenovirus packing materials in HEK293 cells. The adenovirus titer was determined by hole-by-dilution titer assay. The silencing effect of AD-*RPS6KA2-*shRNA in C57BL/6J murine CSPC was validated by real-time PCR and Western-blotting.

### Real-time PCR

Total RNA from cells was extracted using RNAiso Plus reagent (TAKARA, China), according to the manufacturer's instructions. Total RNA was transcribed into cDNA using a PrimeScript RT Master Mix kit (TransGen Biotech, China). The real-time quantitative PCR reaction was performed using the TransStart Tip green qPCR SuperMix kit (TransGen Biotech, China) in an ABI 7500 Real Time PCR System (Applied Biosystems, USA). Primer sequences were detailed in Table S1. The β-actin or GAPDH gene was amplified separately as an internal control to normalise for specific gene expression in the samples. Fold change was calculated using the 2^-DDCt^ method.

### Immunochemistry and immunofluorescence

Cells were fixed with 4% paraformaldehyde for 10 min and permeabilized with 0.5% triton X-100 for 5 min. The knee joints were fixed in 4% paraformaldehyde for 48 h, decalcified in 10% ethylenediamine tetraacetic acid (EDTA, pH 7.4) for 14 d and embedded in paraffin. Paraffin-embedded tissue was cut into 5μm-thick sections. The tissue sections were digested with pepsin (0.25 mg/ml, Sigma) for antigen retrieval. Endogenous peroxidase activity was blocked by incubating the sections in 3% hydrogen peroxide for 10 min (only for immunohistochemistry). The cells and sections were then incubated with 5% bovine serum albumin (BSA, Sigma) and incubated overnight with mouse-anti Type II collagen (Developmental Studies Hybridoma Bank, 1:20, II-II6B3), rabbit-anti Aggrecan (Proteintech, 1:200, 13880-1-AP), rabbit-anti MMP-13 (Abcam, 1:200, ab39012), mouse-anti Ki67 (Abcam, 1:200, ab8191), rabbit-anti CD44 (Abcam, 1:200, ab157107), mouse-anti CD105 (Abcam, 1:200, ab11414), and rabbit-anti Type X collagen (Abcam, 1:200, ab58632), rabbit-anti p-RSK-3 (RD system, 1:200, AF893) diluted with 3%BSA. Then, the cells or sections were incubated with Alexa Flour 488/546-conjugated donkey anti-rabbit/mouse secondary antibody (Invitrogen, USA) for immunofluorescence or HRP goat anti-rabbit/mouse secondary antibodies (KPL, USA) for immunochemistry. Diaminobenzidine tetrahydrochloride (ZSGB-Bio, China) was used for immunochemical staining. The negative control was treated with the same steps without primary antibodies incubation. The images were captured under a microscope (Olympus BX51, Japan). Quantitative analysis was conducted in a blinded fashion with Image-Pro Plus Software.

### Western blotting

Total protein was obtained by lysing human or mouse cells in radioimmunoprecipitation assay (RIPA) buffer containing a protease inhibitor cocktail. The prepared samples were separated on 10% or 8% sodium dodocyle sulfate-polyacrylamide gel electrophoresis (SDS-PAGE) gels and transferred to polyvinylidene difluoride (PVDF) membranes (Millipore, Temecula, CA, USA). After blocking with 3% BSA, the membranes were incubated overnight at 4°C with primary antibodies including mouse-anti Type II collagen (Developmental Studies Hybridoma Bank, 1:100, II-II6B3), rabbit-anti Type I collagen (Proteintech, 1:500, 14695-1-AP), rabbit-anti MMP-2 (Proteintech, 1:500, 10373-2-AP), rabbit-anti Aggrecan (Proteintech, 1:500, 13880-1-AP), rabbit-anti RSK-3 (Proteintech, 1:500, 14446-1-AP), rabbit-anti p-ERK1/2 (Cell Signaling Technology, 1:500, 9101), rabbit-anti p-AKT (Cell Signaling Technology, 1:500, 9271), rabbit-anti AKT (Cell Signaling Technology, 1:500, 9272), rabbit-anti p-JUN (Cell Signaling Technology, 1:500, 9261), rabbit-anti p-RSK-3 (RD system, 1:500, AF893) rabbit-anti CDK-2 (Abcam, 1:500, ab32147) or mouse-anti β-actin (Abcam, 1:2000, ab8226), washed in TBS-Tween 20, and incubated with horseradish peroxidase-coupled anti-rabbit or anti-mouse antibodies (KPL, Gaithersburg, MD, USA), which was detected by chemical luminescence and visualized on a luminescent image analyzer (ImageQuant LAS4000mini, Sweden). Densitometric analyses were performed using Gel-Pro Analyzer software (Media Cybernetics, Rockville, USA).

### Statistical analysis

All data are expressed as the means ± standard deviation. Differences between groups were assessed by independent-samples T test or by one-way analysis of variance (ANOVA) followed by Tukey's Multiple Comparison Test, performed in SPSS 22.0 software. A p < 0.05 was considered statistically significant.

## Results

### 4-ABP enters the cartilage after oral KGN administration

**Figure 1:**
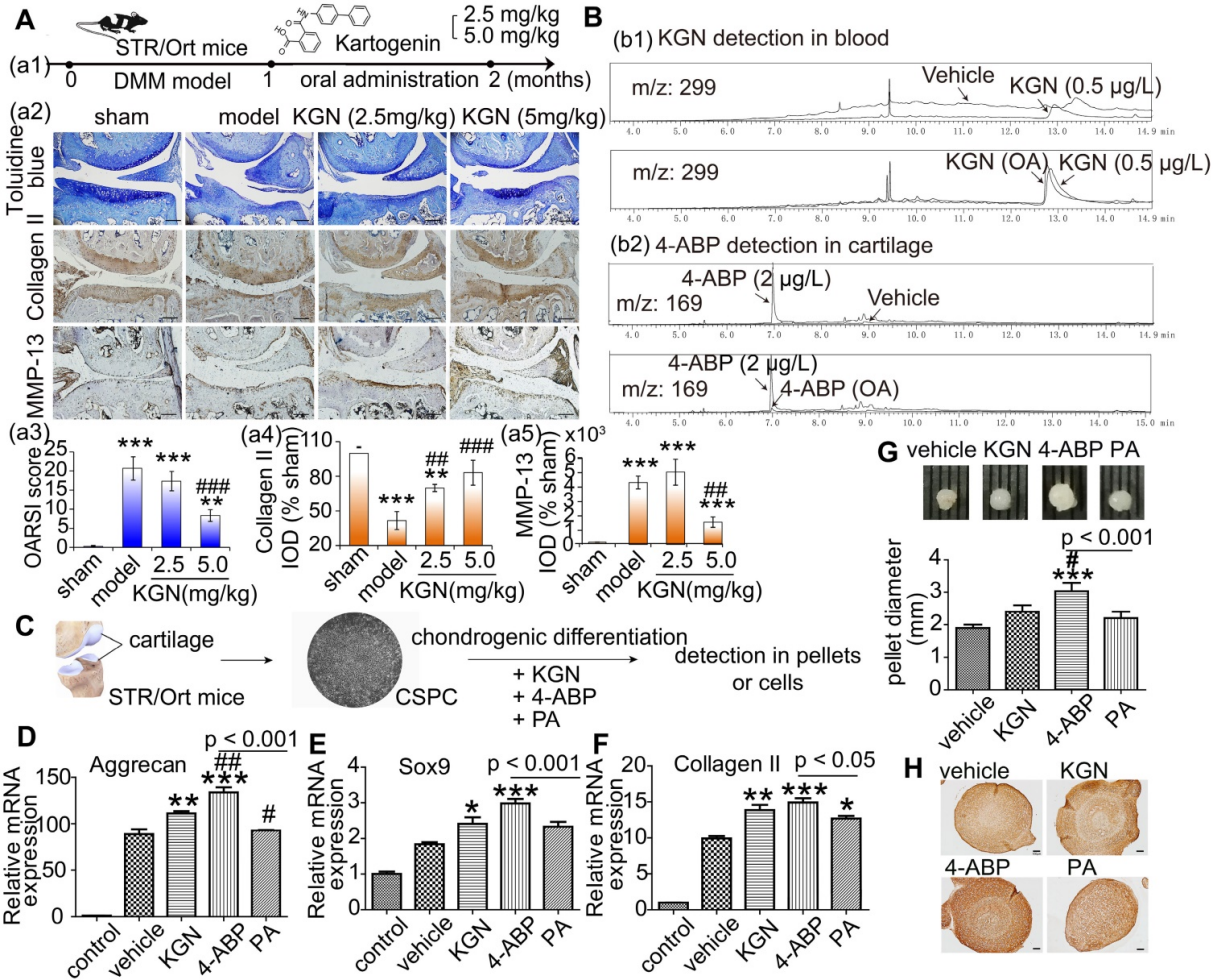
** 4-aminobiphenyl (4-ABP) is found in cartilage after oral kartogenin (KGN) administration and promotes chondrogenic differentiation of cartilage-derived-stem/progenitor cells (CSPC).** (A) Oral KGN administration schedule in destabilization of medial meniscus (DMM)-induced osteoarthritis in STR/Ort mice (a1). Representative images of toluidine blue and immunohistochemical staining of type II collagen and MMP-13 in articular cartilage (a2). Scale bars = 100 μm. OARSI score for osteoarthritis cartilage histopathological assessment (a3-a5). **p < 0.01, ***p < 0.001 versus sham; ^##^*p* < 0.01, ^###^*p* < 0.001 versus model. n = 5 per group. (B) HPLC/MS analysis of KGN and 4-ABP in blood (b1) and cartilage (b2) after 5 mg/kg KGN oral administration (OA) to STR/Ort mice. (C) CSPC were isolated from cartilage and treated with vehicle or 10 μM KGN, 4-ABP or phthalic acid (PA) in the chondrogenic differentiation medium. (D-F) Aggrecan, Sox9 and type II collagen mRNA expression levels (determined by RT-qPCR) at day 21. Pellet diameters (G) and their type II collagen expression (H) at day 21. *p < 0.05, **p < 0.01, ***p < 0.001 versus the vehicle group; ^#^*p* < 0.05 versus the KGN group. n = 6 per group.

KGN administration by intra-articular injection has been the predominant exposure route chosen in previous studies. To facilitate the future application of KGN-based therapy in patients with OA, we examined the effects of oral KGN administration. We found that administering 5 mg/kg KGN daily for one month (Figure 1A-a1) markedly improved cartilage injury in a mouse model of surgical cartilage DMM. Specifically, we found reduced cartilage matrix loss, and increased type II collagen expression (Figure 1A-a2,a3,a4, negative control in Figure S1A and preimmune antibody control in Figure S1B). MMP-13 is a major matrix-degrading enzyme causing cartilage degeneration and its level correlates with the presence of pathological chondrocytes that undergo hypertrophic differentiation in the early stage of OA 33. In the mouse model, MMP-13 expression increased significantly followed by cartilage loss (Figure 1A-a2,a5, , negative control in Figure S1A and preimmune antibody control in Figure S1B). Although giving 2.5 mg/kg KGN could reduce cartilage loss, but not decreased the number of pathological chondrocytes with a high level of MMP-13 in the cartilage (Figure 1A-a2,a5), While 5 mg/kg KGN could markedly protect cartilage against DMM injury with integrated cartilage and low expression of MMP-13. So oral administration of KGN had protective effects in OA mouse model.

To confirm whether KGN could enter the cartilage, we used HPLC/MS methods to assess KGN levels in the blood and cartilage after oral administration. Surprisingly, while KGN was only found in blood (Figure 1B-b1), we detected the hydrolysate 4-ABP (about 0.28 ng in cartilage from one mice) in the cartilage (Figure 1B-b2). Moreover, KGN and 4-ABP (about 0.34 ng in blood from one mice) were both found in the blood after single intravenous KGN injection (Figure S2). These data suggested that 4-ABP could enter the cartilage, where it might have a role in ameliorating DMM-induced cartilage injury.

### 4-ABP is a stronger inducer of chondrogenic differentiation than KGN

KGN can induce MSC chondrogenic differentiation 10, but this has not been shown for 4-ABP. Interestingly, we found that after treating rat BMSC (identified in Figure S3) for 3 days with KGN, 4-ABP or PA, 4-ABP also significantly promoted chondrocyte differentiation as demonstrated by increased *type II collagen*, *lubricin* and *aggrecan* gene expression compared with KGN (Figure S4). These effects occurred in a dose-dependent manner. Conversely, PA did not induce chondrogenic differentiation. Our previous study indicated that CSPC from STR/Ort mice, a spontaneous OA model, have a low capacity to proliferate and differentiate 30. Here, exposing CSPC (Figure 1C) to chondrogenic medium conditions resulted in increased *aggrecan*, *Sox9* and *type II collagen* expression in the vehicle group, while supplement with 4-ABP further elevated these gene's expression (Figure 1D-F), cellular pellet diameters by up to 30% (Figure 1G) and type II collagen expression (Figure 1H), indicative of 4-ABP on promoting chondrogenic differentiation and proliferation of CSPC and its potential role on OA treatment.

**Figure 2:**
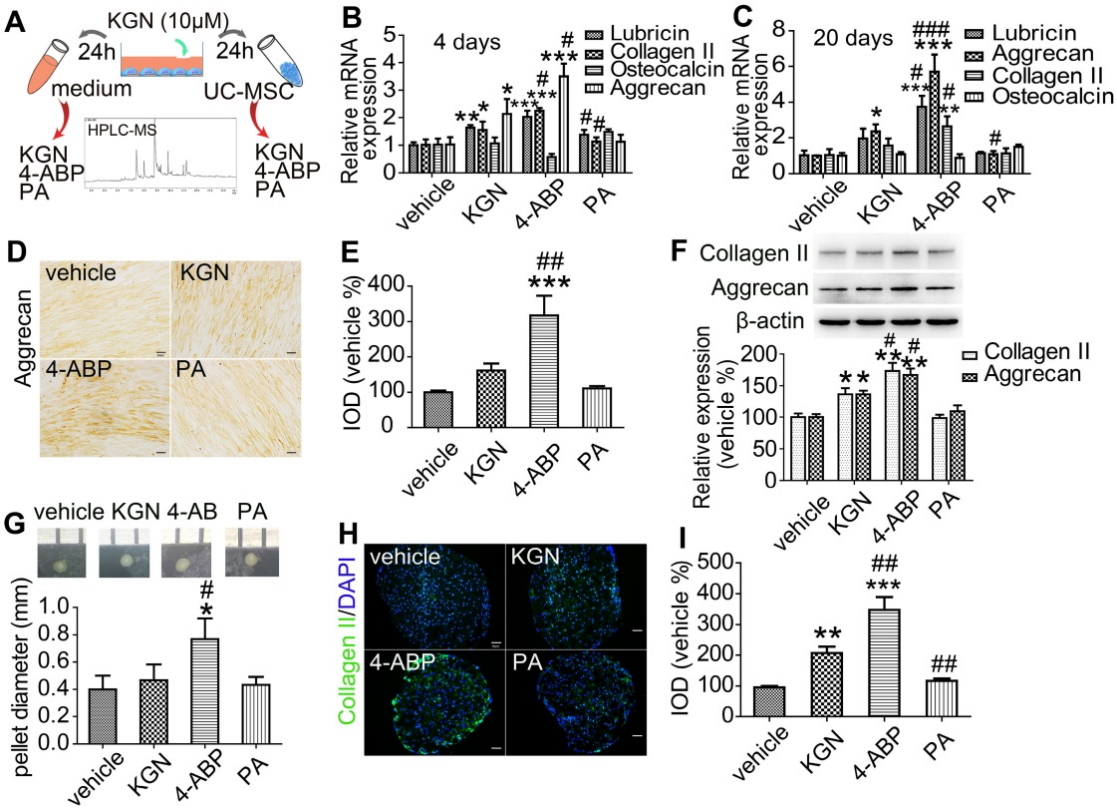
** 4-aminobiphenyl (4-ABP) induces chondrogenic differentiation of umbilical cord mesenchymal stem cells (UC-MSC).** (A) HPLC/MS analysis of kartogenin (KGN), 4-ABP and phthalic acid (PA) in UC-MSC culture medium and UC-MSC after KGN (10 μM) treatment for 24 h. (B-F) Chondrocyte-specific gene and protein expression after KGN, 4-ABP or PA treatment for 4 days (B) and 20 days (C-F) in UC-MSC under monolayer culture. UC-MSC pellets diameters (G) and type II collagen expression and analysis (H,I) after chemical treatment for 20 days. Scale bars = 50 μm. *p < 0.05, **p < 0.01, ***p < 0.001 versus vehicle; ^#^*p* < 0.05, ^##^*p* < 0.01, ^###^*p* < 0.001 versus KGN. n = 6 per group.

We next aimed to determine whether the cleavage of KGN was occurring *in vitro* and whether the products were naturally produced and had the same effects as above by treating the human UC-MSC (identified in Figure S5) with the purified products. We exposed UC-MSC to KGN for 24 h and then detected cleavage into 4-ABP and PA by HPLC/MS (Figure 2A and Figure S6). This finding suggests that KGN-induced chondrogenic differentiation might be mediated by 4-ABP. Evidence for stronger 4-ABP-mediated lineage-specific differentiation compared to KGN was further confirmed by time-dependent increases in chondrocyte-specific gene and protein levels, including lubricin, type II collagen and aggrecan under monolayer culture (Figure 2B-F, , negative control in Figure S1A and preimmune antibody control in Figure S1B). The peripheral cells around the pellets were directly affected by the chemicals. 4-ABP-treated pellets had a higher expression of type II collagen in the periphery of pellets than the other groups, which indicated the stronger stimulation of chondrogenic cell differentiation by 4-ABP (Figure 2H,I). Moreover, neither KGN, 4-ABP nor PA altered *osteocalcin* gene expression, which is a marker of chondrocyte calcification, in rat BMSC or human UC-MSC (Figure S4 and Figure 2B,C). Finally, we found that 4-ABP treatment significantly increased the size of CSPC or human UC-MSC pellets compared to PA and KGN (Figure 1G and Figure 2G). Taken together, 4-ABP induced both chondrocyte-specific differentiation and proliferation of CSPS and UC-MSC more effectively than KGN.

### 4-ABP ameliorates cartilage injury in OA mice and CD44^+^/CD105^+^ cell loss

**Figure 3:**
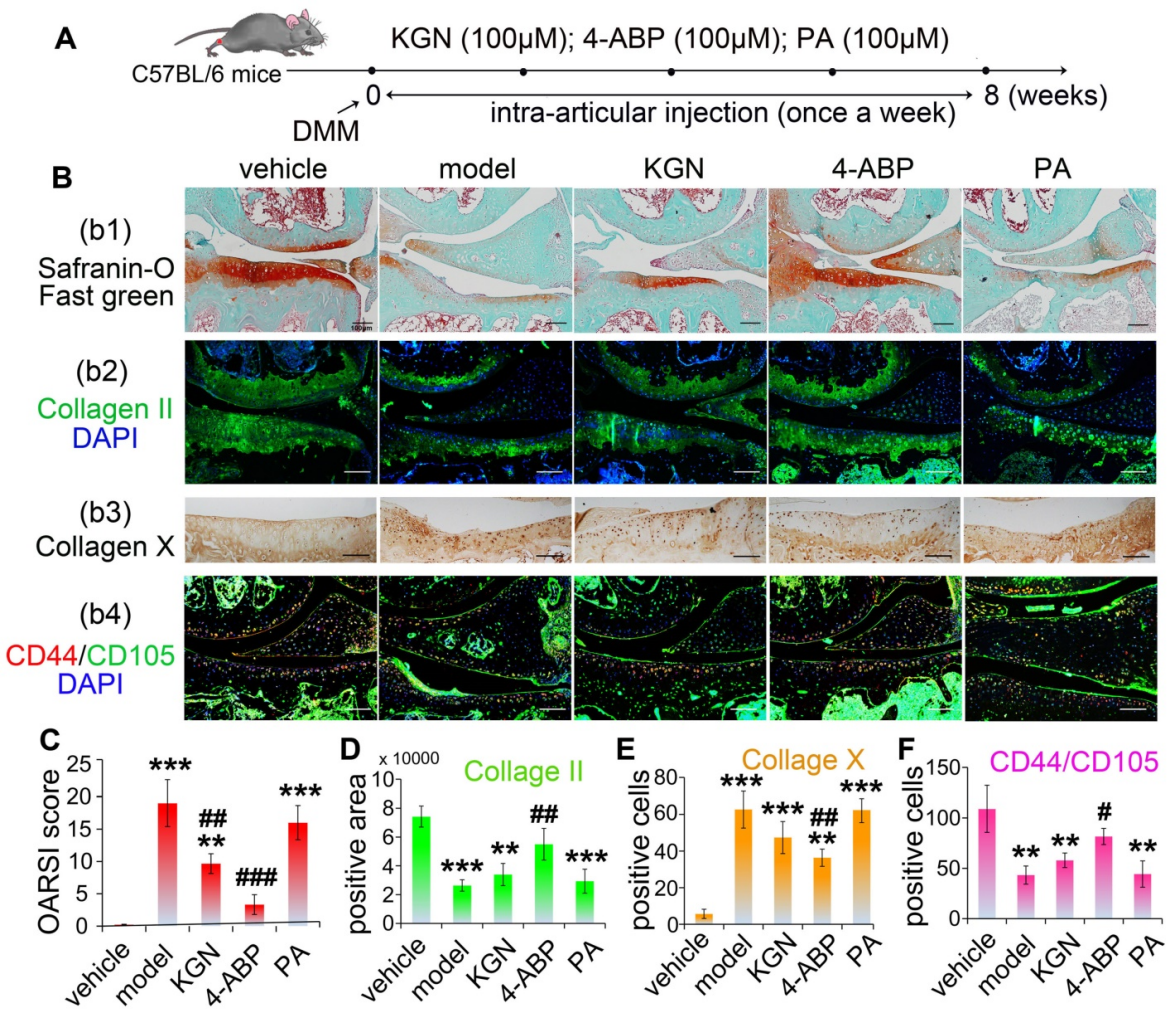
** 4-aminobiphenyl (4-ABP) promotes cartilage repair in the destabilization of medial meniscus** (**DMM) surgery-induced osteoarthritis mouse model.** (A) Treatment schedule. (B) Mouse knee joints were stained with safranin-O/fast green (b1), type II collagen (b2), type X collagen (b3) and CD44/CD105 (b4). Nuclei were counterstained with DAPI where indicated. (C-F) Statistical analysis of the data shown in (B). Scale bars = 100 μm. **p < 0.01, ***p < 0.001 versus vehicle; ^#^*p* < 0.05, ^##^*p* < 0.01, ^###^*p* < 0.001 versus model. n = 6 per group.

We next focused our analyses on whether the cleavage of KGN was occurring in cartilage and assessed the *in vivo* effects of KGN, 4-ABP or PA in OA mice. First, we were able to detect both KGN and 4-ABP (about 2.24 ng in cartilage from one mice) in the cartilage after intra-articular injection of KGN in C57BL/6J mice (Figure S7). Next, we induced DMM injury in C57BL/6J mice and then evaluated KGN, 4-ABP or PA efficiency *in vivo* after injection into the articular cavity (Figure 3A). After intra-articular administration of 4-ABP once-weekly for 8 weeks, histological observations and OARSI scores revealed marked increases in cartilage matrix production and improvements in DMM injury compared to vehicle and KGN-treated mice (Figure 3B-b1,b2,C,D). However, no obvious improvement was observed after PA treatment compared with the model group. Additionally, 4-ABP treatment was associated with significantly decreased hypertrophic chondrocytes labeled with type X collagen (Figure 3B-b3,E, , negative control in Figure S1A and preimmune antibody control in Figure S1B) and increased numbers of CD44^+^/CD105^+^ CSPC compared to model mice (Figure 3B-b4,F, , negative control in Figure S1A and preimmune antibody control in Figure S1B). Collectively, these *in vitro* and *in vivo* data strongly supported that 4-ABP promoted CSPC and BMSC chondrocytic differentiation, and increased the number of cartilage-resident MSC, which helped repair damaged joints.

### 4-ABP promotes human MSC proliferation by activating the PI3K-Akt signaling pathway

**Figure 4:**
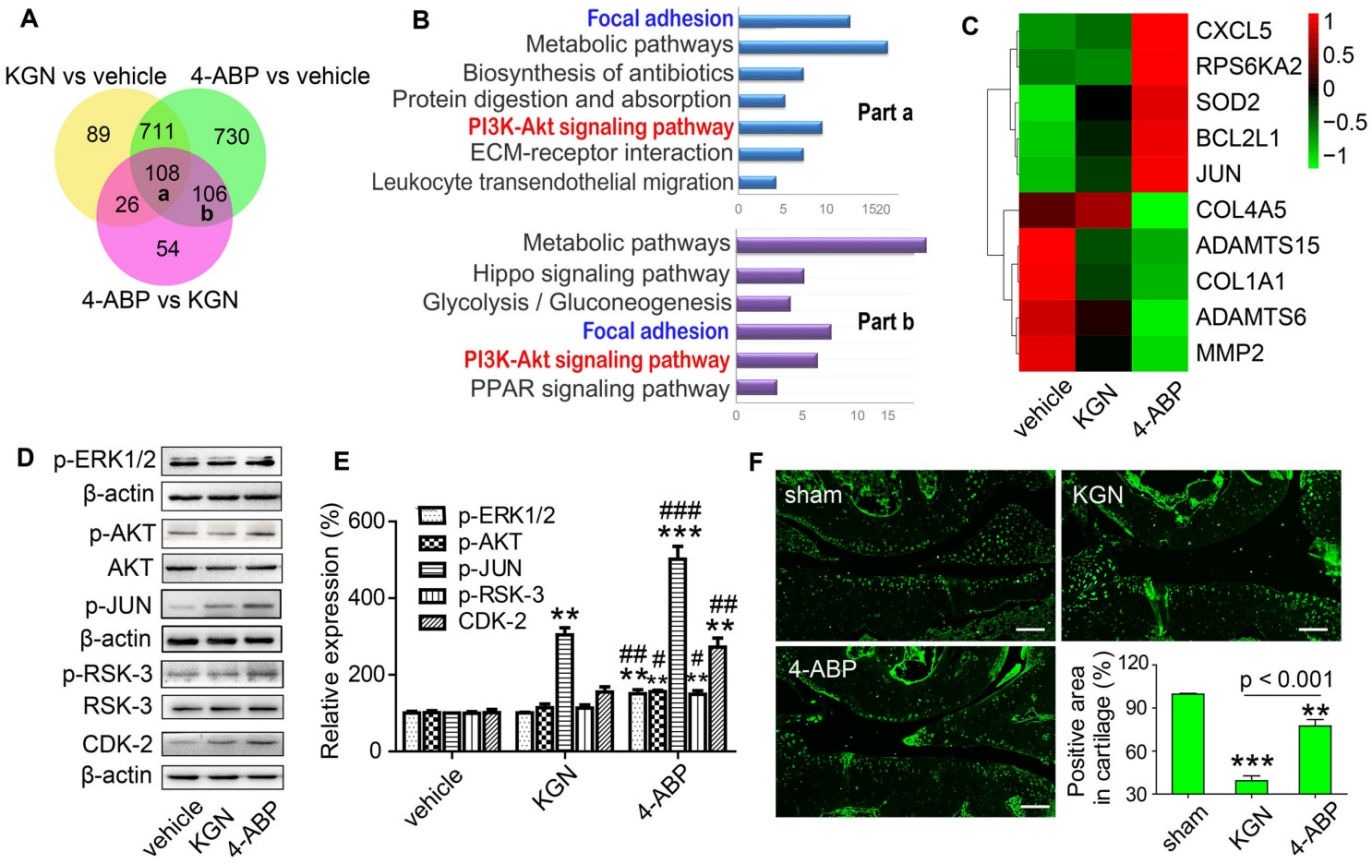
** 4-aminobiphenyl (4-ABP) activates the PI3K-Akt pathway in umbilical cord mesenchymal stem cells (UC-MSC)**. (A-C) Bioinformatic Venn diagram (A), KEGG (B) and Heatmap (C) analyses of the key molecules and signaling pathways differentially regulated in UC-MSC treated with kartogenin (KGN) or 4-ABP (10 μM) for 3 days based on transcriptome analysis. (D-E) p-ERK1/2, p-AKT, p-JUN, p-RSK-3 and CDK-2 protein expression in UC-MSC treated with vehicle, KGN or 4-ABP (10 μM) for 3 days. **p < 0.01, ***p < 0.001 versus vehicle; ^#^*p* < 0.05, ^##^*p* < 0.01, ^###^*p* < 0.001 versus KGN. n = 3 per group. (F) p-RSK-3 expression in cartilage from C57BL/6J mice subjected to destabilization of medial meniscus surgery. Scale bars = 100 μm. **p < 0.01, ***p < 0.001 versus sham. n = 5 per group.

To elucidate the biological mechanisms of 4-ABP in cartilage repair, we performed RNA sequencing to examine the transcriptome profiles of UC-MSC treated with KGN or 4-ABP daily for 3 days. By Venn analysis (Figure 4A), we found that the intersection of the differentially expressed (DE) RNAs between KGN versus vehicle, 4-ABP versus vehicle and 4-ABP versus KGN (part a in Figure 4A and Figure S8A) was associated with the enhanced chondrogenic capacity caused by KGN or 4-ABP. These upregulated and downregulated mRNAs could be grouped into multiple signaling pathways, namely metabolic pathways, focal adhesion and the PI3K-Akt signaling pathways (Figure 4B). We thus constructed a network based on these DE RNAs (Figure S8A), which showed that the key RNAs, such as MMP-2 (matrix degradation enzyme) and type I collagen (a fibrocartilage marker) 34, were down-regulated (Figure S9), indicative of cartilage protection. Moreover, part b in the Venn diagram (Figure 4A and Figure S8B) represented the specific action of 4-ABP, which was different from both KGN and vehicle groups. Specifically, we identified that PI3K-Akt and focal adhesion signaling pathways (Figure S8B) and some core RNAs, such as *JUN* and *RPS6KA2*, were up-regulated and enriched, which might contribute to the enhanced cell proliferation and increased pellet diameters caused by 4-ABP.

Next, we verified the changes observed at the RNA and protein level in the PI3K-Akt signaling pathway. After treating UC-MSC with KGN or 4-ABP for 4 days, we found that 4-ABP significantly increased p-ERK1/2, p-AKT, p-JUN, p-RSK-3 (encoded by *RPS6KA2*) and CDK-2 protein expression (important components of the PI3K-Akt pathway) compared with KGN treatment (Figure 4D,E). Finally, to confirm the results *in vivo*, we administered DMM mice with 4-ABP or KGN via intra-articular injection. Here, we also found that p-RSK-3 levels were elevated markedly in the cartilage of 4-ABP versus to KGN mice (Figure 4F). In summary, 4-ABP could increase the molecular expressions in the PI3K-Akt signaling pathway, which contributed to the MSC proliferation.

### RSK-3, a key regulator of 4-ABP in promoting CSPC proliferation

**Figure 5:**
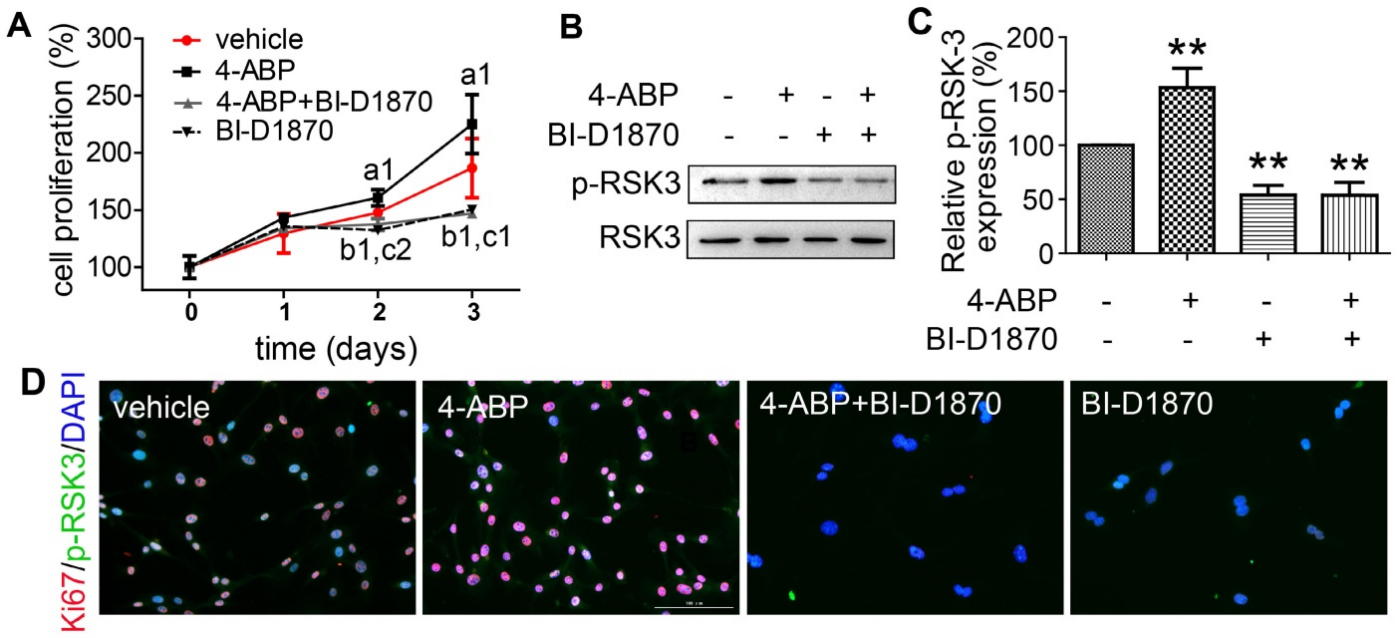
** The RSK-3 inhibitor BI-D1870 suppresses 4-aminobiphenyl (4-ABP)-enhanced cartilage-derived-stem/progenitor cell (CSPC) proliferation.** (A-D) CSPC from C57BL/6J mice were treated with vehicle, 4-ABP (10 μM), 4-ABP (10 μM) plus BI-D1870 (10 μM) or BI-D1870 (10 μM) alone. (A) Cell proliferative ability of CSPC was monitored 0-3 days after treatment, by CCK-8 method. ^a1^*p* < 0.05 4-ABP versus vehicle; ^b1^*p* < 0.05 4-ABP+BI-D1870 versus vehicle; ^c1^*p* < 0.05, ^c2^*p* < 0.01 BI-D1870 versus vehicle. n = 8 per group. (B) p-RSK-3 protein expression after chemical treatment for 3 days. (C) Relative p-RSK3 expression to total RSK3 is shown. **p < 0.01 versus vehicle, n = 3. (D) Ki67 (red) and p-RSK-3 (green) protein expression in CSPC after BI-D1870 and 4-ABP treatment. Nuclei are counterstained with DAPI. n = 3.

**Figure 6:**
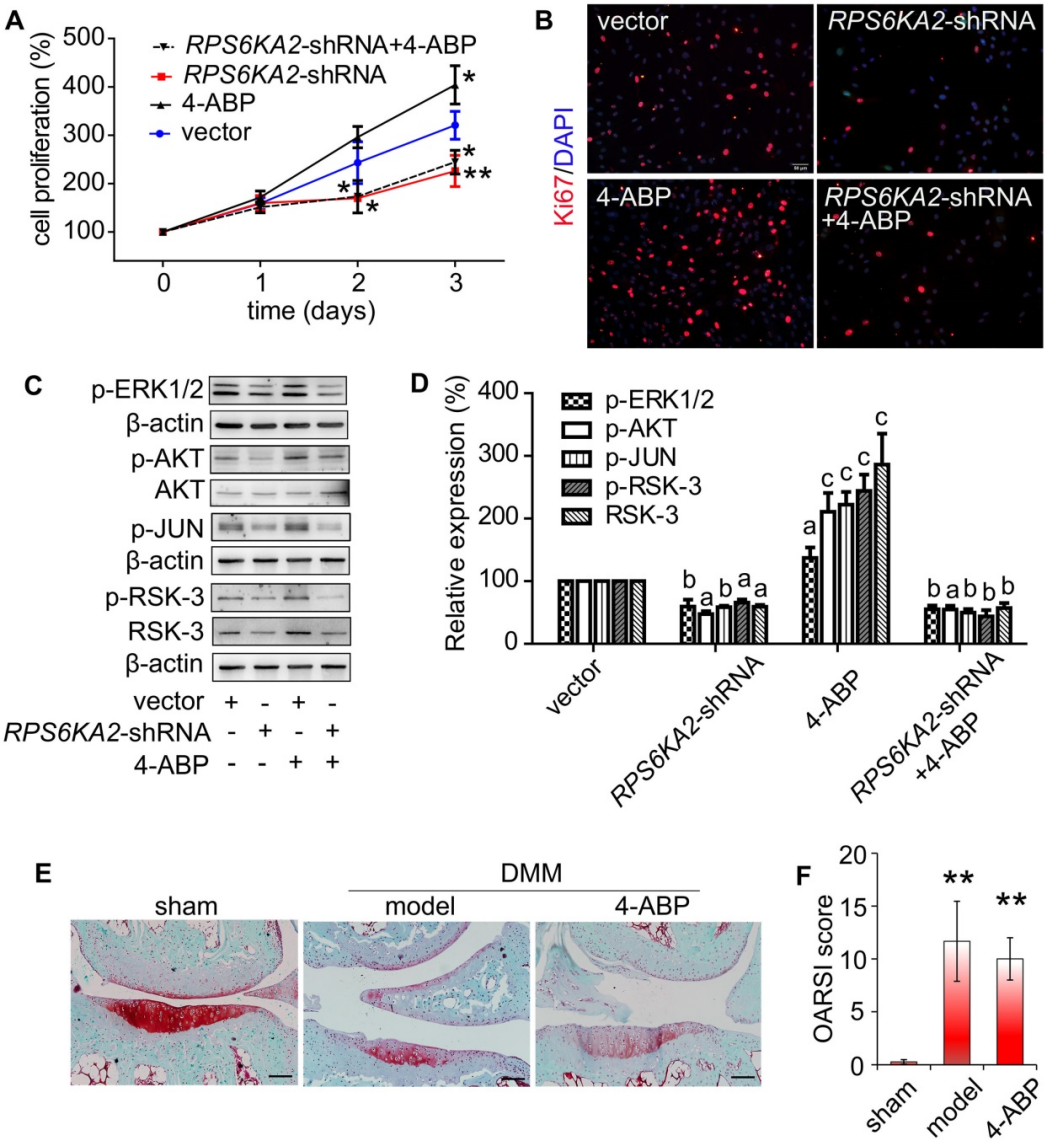
** RSK-3 knockdown blocks 4-aminobiphenyl (4-ABP)-enhanced cartilage-derived-stem/progenitor cell (CSPC) proliferation and OA protection.** (A-B) CSPC proliferation after RSK-3 knockdown (*RPS6KA2*-shRNA) or *RPS6KA2*-shRNA plus 4-ABP treatment, determined by CCK-8 assay (A) and Ki67 labeling (B). Nuclei are counterstained with DAPI. Effects of 4-ABP on protein expression of molecules involved in the PI3K-Akt pathway in *RPS6KA2*-shRNA-treated CSPC (C) and statistical analysis (D). ^a^*p* < 0.05, ^b^*p* < 0.01, ^c^*p* < 0.001 versus vector control. n = 3 per group. Safranin O-Fast green staining of cartilage lesions in RSK-3 knockout mice after destabilization of medial meniscus (DMM) surgery followed by intra-articular 4-ABP (100 μM) injection for 4 weeks (once a week) (E) and OARSI score (F). Scale bars = 100 μm. **p < 0.01 versus sham. n = 5.

To confirm a key role for RSK-3 in 4-ABP-mediated MSC proliferation, we exposed CSPC from C57BL/6J mice to an RSK-3 inhibitor, BI-D1870. RSK-3 inhibition significantly inhibited RSK-3 phosphorylation and CSPC proliferation, which was not reversed upon 4-ABP treatment (Figure 5). We then used the RPS6KA2-shRNA to knockdown the protein expression of RSK-3. Here, 4-ABP markedly increased the number of un-treated CSPC, but not RSK-3-knockdown-treated CSPC (Figure 6A,B), which was associated with inhibition of the PI3K-Akt pathway, exhibiting down-regulated p-ERK1/2, p-AKT, p-JUN and p-RSK-3 (Figure 6C,D).

Finally, we used RSK-3-knockout mice to determine whether RSK-3 was the target of 4-ABP *in vivo*. DMM surgery in these knockout mice caused an obvious loss of cartilage matrix that could not be improved upon 4-ABP treatment (Figure 6E,F). These data implied that RSK-3 was a key regulator of 4-ABP activity on MSC proliferation and cartilage injury repair in OA.

## Discussion

With the initiative of developing easy and convenient prophylactic techniques for OA patients, this study aimed to test the action of KGN on cartilage regeneration by oral administration and the further pharmacological mechanism. After confirming that oral KGN administration could attenuate OA injury, we wished to confirm whether KGN could actually disperse within the cartilage. While we detected KGN in the blood by HPLC/MS, to our surprise, new chromatographic peaks in the blood were detected (data not shown) and no KGN was found present in cartilage after oral administration of KGN. The finding raised the question as to what happens to KGN upon entering the blood stream.

Previous studies have shown that amide bond cleavage can be catalyzed by various enzymes, such as serine proteases, metalloproteases, cysteine proteases and aldehyde oxidase 35, 36. Here, we hypothesized that KGN might be decomposed into 4-ABP and PA after cellular exposure. Indeed, HPLC/MS detected 4-ABP in cartilage after oral, intravenous or intra-articular KGN administration. Thus far, few compounds were actually detectable in cartilage or synovial fluid after systemic or local delivery 37. The presence of 4-ABP within cartilage must exert certain effects on cartilage regeneration. Most interestingly, 4-ABP showed a higher capacity to ameliorate the OA injury than KGN in terms of enhancing chondrogenic differentiation and MSC proliferation. While many studies have reported that KGN promotes MSC chondrogenesis and proliferation 10, 12-16, the current study has confirmed that these effects are mediated by 4-ABP. Consequently, KGN may have served as a pro-drug in previous studies, with its action limited by the rate of amide hydrolysis. Studies that focus on improving the KGN structure for specific cleavage in blood or cartilage are now warranted.

The STR/Ort mouse is a spontaneous OA model exhibiting age-dependent cartilage degeneration of the joint, similar to human OA 38, 39. Our previous study indicated that the limited proliferation and chondrogenic differentiation capacity of CSPC contributed to the OA of STR/Ort mice 30. So the STR/Ort mouse and their CSPC were suitable for evaluating the regenerative effects of chemicals in OA. In this study, DMM-induced cartilage injury on this OA-susceptible murine background accelerated the OA progress, and we found the improvement of injured cartilage caused by 4-ABP, which indicated the potential actions of 4-ABP on promoting cartilage regeneration. C57BL/6J mice were generally used as the normal control of STR/Ort mice 40, 41. Moreover, the RSK-3^-/-^ mice were backcrossed to C57BL/6J mice in this study. So C57BL/6J mice were used to furtherly tested the effects of 4-ABP in OA. Chemical induction of endogenous MSC seems to be a promising strategy to selectively modulate MSC functionality 3, 42. Besides mouse CSPC, rat BMSC and human UC-MSC were used in this study to test the effects of chemicals on stem cells. Different species and different types of stem cells could sufficiently confirm the actions of chemicals on proliferation and chondrogenic differentiation in rodents and human beings. Furthermore, the effects of 4-ABP on BMSC and UC-MSC suggested their possible application of exogenous stem cells combined with 4-ABP in OA treatment. So 4-ABP had the potentials for OA therapy by affecting the native joint-resident CSPCs and culture-expanded stem cells.

In the current study, 4-ABP significantly stimulated chondrocyte-specific gene expression of CSPC, implying that 4-ABP might be a chemical modifier of MSC properties such that they were induced to proliferate and differentiate. The transcriptomic analyses of KGN- or 4-ABP-treated UC-MSC demonstrated that 4-ABP could activate the PI3K-Akt pathway. PI3Ks comprise a family of lipid kinases implicated in regulating a wide array of physiological processes, notably the proliferation, cell survival, cell migration and trafficking 43, 44. PI3K-dependent signaling also contributes to embryonic stem-cell self-renewal and pluripotency 45, 46. Importantly, the PI3K-Akt pathway is involved in regulating chondrocyte proliferation and differentiation 47 and is a key regulator of terminal chondrocyte differentiation in both embryonic and adult chondrogenesis 48. JNK is a key player in the PI3K-Akt pathway and is related to the re-organization of the actin cytoskeleton, which is essential for chondrogenesis of MSC 49, 50. Consistently, our bioinformatic analyses of transcriptomic data indicated that 4-ABP activated the PI3K-Akt pathway and promoted the expression of downstream molecules, such as cell cycle protein CDK-2.

Bioinformatic network analysis also suggested that the gene *RPS6KA2* (encoding RSK-3) was a potential 4-ABP target. RSK-3 is part of a family of highly conserved Ser/Thr kinases and consists of four human isoforms (RSK1-4) that regulates diverse cellular processes, such as cell growth, motility, survival and proliferation 51. The RSK isoforms are directly activated by ERK1/2 or PDK1 via the Ras-ERK/MAPK signaling cascade upon the stimulation of growth factors, hormone, neurotransmitters or chemokines 52. In this study, *in vitro* and *in vivo* analyses showed that 4-ABP was unable to promote MSC proliferation in *RPS6KA2-*knockdown cells or ameliorate OA in *RPS6KA2*-knockout mice, confirming that RSK-3 was the key 4-ABP target.

While it was exciting to report that 4-ABP stimulated MSC proliferation and chondrogenic differentiation and speculated that 4-ABP might be a potential drug for OA therapy, some previous studies had suggested that 4-ABP was a DNA-reactive carcinogen and might cause bladder cancer 53-55. 4-ABP is a reported carcinogenic component of tobacco smoke 56. However, it is further proved that 4-ABP toxicity is induced by its metabolic activation to form a reactive electrophile by N-hydroxylation and N-esterification reaction; this electrophile covalently binds to DNA, principally to deoxyguanosine, causing an increased rate of DNA mutations 53. The N-hydroxylation reaction is catalyzed to form N-hydroxy-ABP largely by members of the cytochrome P450 1A family, particularly CYP1A2 55. Then, N-acetyltransferases activate the N-acetylation of N-hydroxy-ABP to produce DNA adducts, or undergoes detoxification via O-acetylation 54, 55. This is to say that the liver exposure is essential for 4-ABP activation and differences in organ specificity of 4-ABP toxicities are thought to be due to organ/tissue metabolic activation versus inactivation 54, 55. Intra-articular injection, but not any means of systemic administration, therefore, seems to be the most effective and safest route for 4-ABP administration as it avoids liver metabolism and the production of N-hydroxy-ABP. However, further investigations are needed before 4-ABP can be considered as an OA therapeutic. At first, it is important to determine whether the key enzymes that activate 4-ABP, such as CYP1A2 and N-acetyltransferases, are not expressed in cartilage. Secondly, it must be tested whether 4-ABP can be catalyzed into DNA adducts in cartilage. Lastly, the optimal 4-ABP dosage and dosing regimen must be determined based on balancing its efficacy with safety. Safety evaluations are becoming essential in order to translate the 4-ABP action into the clinical use. This study also raises the similar issue to KGN, the broadly studied parent chemical, and the safety evaluation is also urgently required even if KGN is to be applied in cartilage regeneration.

Although 4-ABP was known as a carcinogen and much work remained prior to the application of 4-ABP in clinical practice. However, the genes and signal pathways identified as 4-ABP targets provided more options for fate-decision regulation of stem cells. (1) The key pathways (such as PI3K-Akt, Hippo and PPAR pathways) and RNAs (such as RPS6KA2, JUN, VEGFA and PTGS1) may influence stem cell proliferation and differentiation, and are strongly associated with OA. Targeting these core mRNAs or their encoding proteins may improve cartilage regeneration by increasing CSPC proliferation and chondrogenic differentiation. (2) In this study, RSK-3 was verified as a target of 4-ABP to promote CSPC proliferation. So the further research may explore the interaction between the RSK-3 and 4-ABP through the computer calculation to predict the possible structure locations of 4-ABP on RSK-3, and find the structure-function relationship, which may support the new chemical design for targeting RSK-3.

## Conclusion

In summary, we demonstrate that KGN stimulates chondrogenic differentiation of MSC via its lytic product 4-ABP. 4-ABP can distribute to cartilage and promote MSC and CSPC proliferation by activating the PI3K-Akt pathway. The 4-ABP action particularly relies on RSK-3 as a key regulator. This study provides novel insights into several aspects of cartilage regeneration, including the effective component and safety concern of using KGN for cartilage regeneration, the future need to modify KGN or 4-ABP chemical structures in order to avoid their potential toxicity and enhance their efficacy. Most interestingly, modulating the RSK-3 function may be an effective strategy in promoting cartilage regeneration in OA and other joint diseases.

## Abbreviations

KGN: Kartogenin
OA: osteoarthritis
4-ABP: 4-aminobiphenyl
PA: phthalic acid
MSC: mesenchymal stem cells
BMSC: bone marrow stromal cells
CSPC: cartilage-derived-stem/progenitor cells
FBS: Fetal bovine serum
DMM: destabilization of medial meniscus
OARSI: Osteoarthritis Research Society International
UC-MSC: umbilical cord mesenchymal stem cells
HPLC-MS: High performance liquid chromatography-mass spectrometry
ESI: electro spray ionization
CCK-8: Cell Counting Kit-8
shRNA: short hairpin RNA
EDTA: ethylenediamine tetraacetic acid
RIPA: radioimmunoprecipitation assay
SDS-PAGE: sodium dodocyle sulfate-polyacrylamide gel electrophoresis
PVDF: polyvinylidene difluoride
ANOVA: one-way analysis of variance
DE: differentially expressed
