# Supplementary material for: Kartogenin hydrolysis product 4-aminobiphenyl distributes to cartilage and mediates cartilage regeneration
Source: Theranostics. 2019 Sep 21;9(24):7108–21. doi: 10.7150/thno.38182 (PMC6831301; doi:10.7150/thno.38182)
Supplement: Supplementary file 1 — Supplementary figures and tables. [file thnov09p7108s1.pdf]

Supplwmental results

Table S1 Primer sequences for RT-q PCR

Name	Sequence (5’ to 3’)
Aggrecan (mice)	TTCCACCAGTGCGATGCAG
	TGGTGTCCCGGATTCCGTA
Sox9 (mice)	CAGCAAGACTCTGGGCAAG
	TCCACGAAGGGTCTCTTCTC
Collagen II (mice)	GGGCTCCAATGATGTAGAGATG
	CCCACTTACCAGTGTGTTTCG
β-actin (mice)	AGCCATGTACGTAGCCATCC
	CTCTCAGCTGTGGTGGTGAA
Lubricin (human)	CGCTGCTTTGAGTCCTTCGAG
	CCTGAAGGTGGAGGTGCTTTC
Aggrecan (human)	CTAGTGGACTCCCTTCAGGAAC
	CGCTAAGCTCAGTCACTCCAG
Collagen II (human)	ACCCTGAGTGGAAGAGTGGAG
	CTTGGGAACGTTTGCTGGATTG
Osteocalcin (human)	CCCACCTGCACAGTACTCC
	ACTGTGGTCTTGCTGGCTTTG
GAPDH (human)	TCTGACTTCAACAGCGACACC
	CTGTTGCTGTAGCCAAATTCGT
Lubricin (rat)	CGCTGCTTTGAGTCCTTCGAG
	CCTGAAGGTGGAGGTGCTTTC
Aggrecan (rat)	CTAGTGGACTCCCTTCAGGAAC
	CGCTAAGCTCAGTCACTCCAG
Collagen II (rat)	ACCCTGAGTGGAAGAGTGGAG
	CTTGGGAACGTTTGCTGGATTG
*Osteocalcin (*rat*)*	CCCACCTGCACAGTACTCC
	ACTGTGGTCTTGCTGGCTTTG
GAPDH (rat)	TCTGACTTCAACAGCGACACC
	CTGTTGCTGTAGCCAAATTCGT

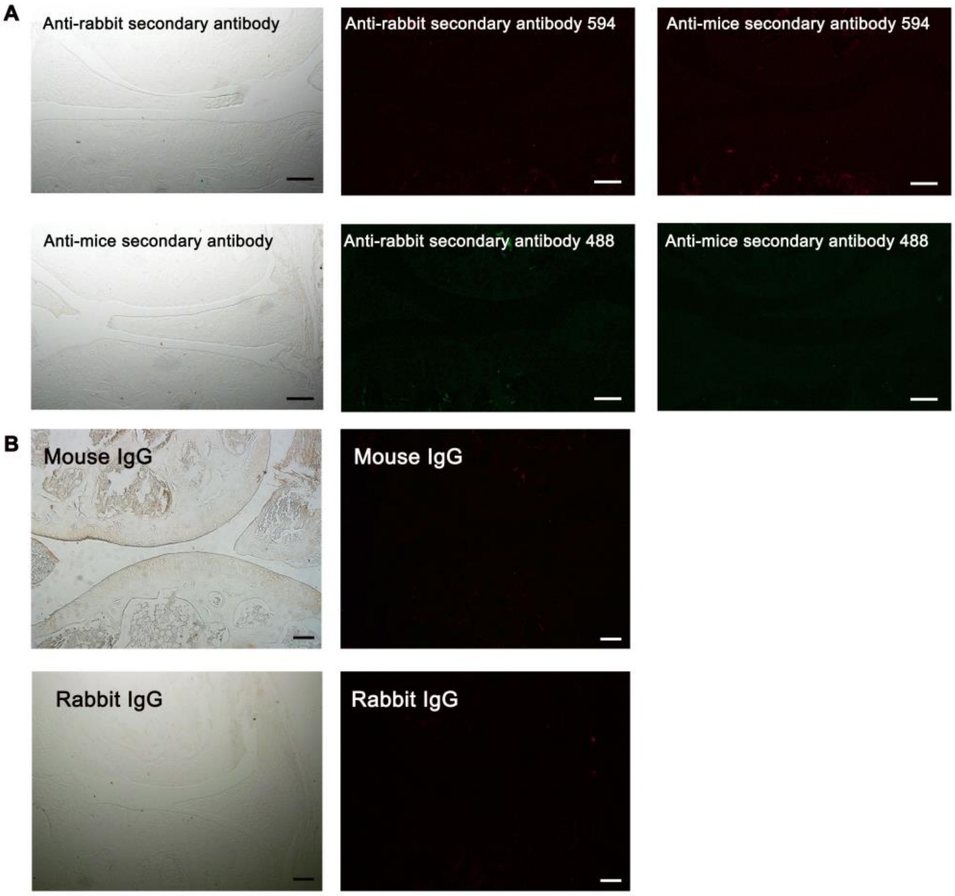

Figure S1 The control for the primary immune antibodies in the articular cartilage slices. (A) The slices were treated with the same methods of immunochemistry and immunofluorescence without primiray antibodies. (B) The slices were treated with the preimmune antibodies (mouse IgG and rabbit IgG, purchased from Proteintech) instead of the primary immune actibodies, and the other steps were the same as immunochemistry and immunofluorescence. Scale bar = 100 μm.

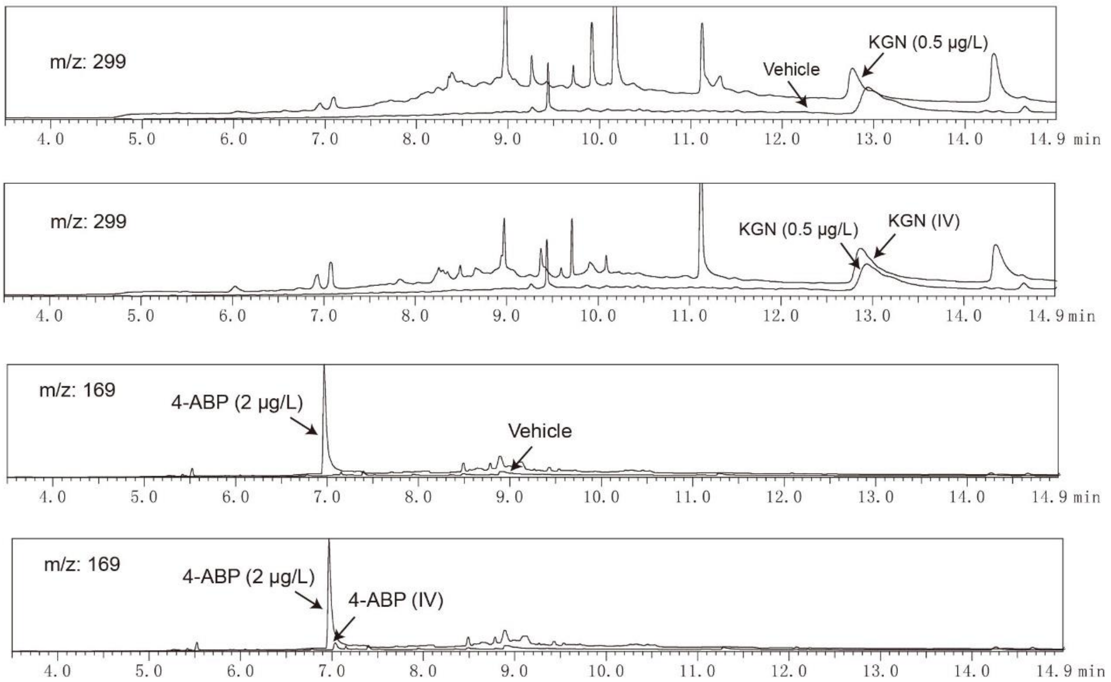

Figure S2 HPLC-MS analysis of blood 24 h after single intravenous injection of kartogenin (KGN) (2.5 mg/kg) in STR/Ort mice.

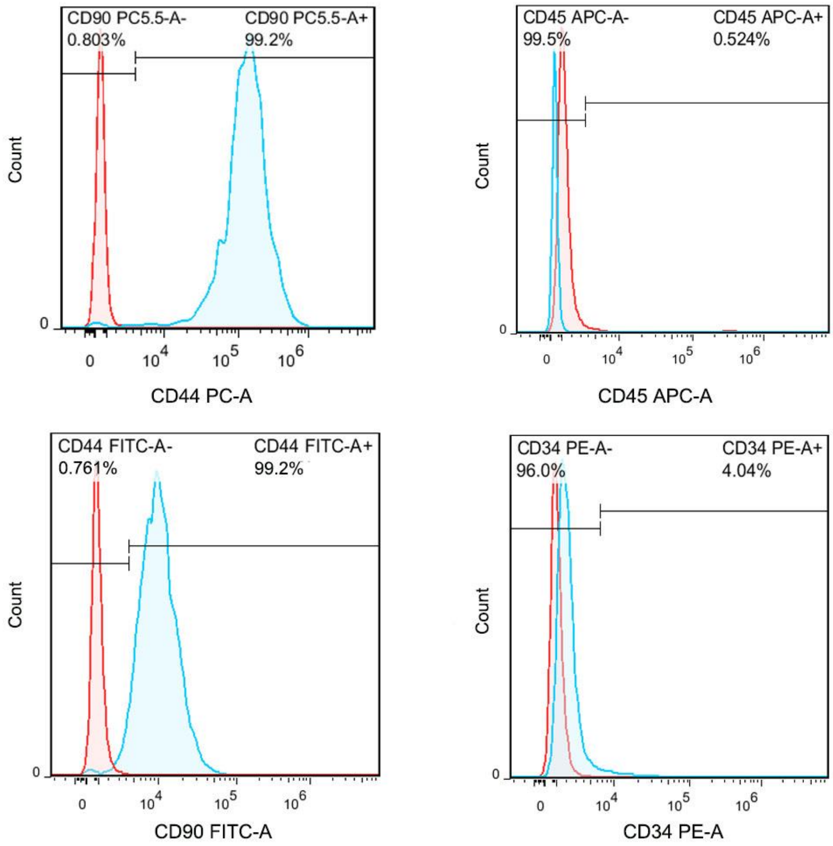

Figure S3 Identification of rat bone marrow mesenchymal stem cells by detecting the positive CD44 and CD90, and negative CD45 and CD34 markers by flow cytometry.

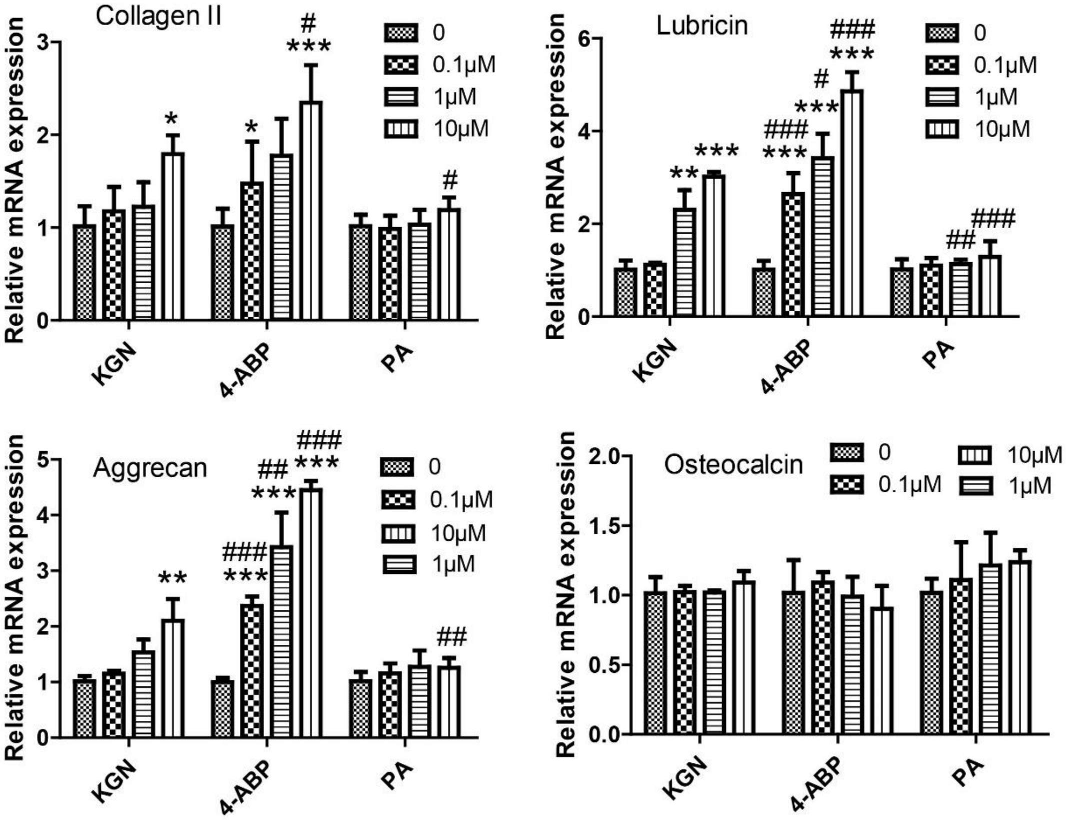

Figure S4 Effects of KGN, 4-ABP and PA on *Collagen II*, *Lubricin*, *Aggrecan* and *Osteocalcin* expression in rat bone marrow mesenchymal stem cells after treatment for 3 days. $^{*}p < 0.05$, $^{**}p < 0.01$, $^{***}p < 0.001$ vs. 0 μM group. $^{\#}p < 0.05$, $^{\#\#}p < 0.01$, $^{\#\#\#}p < 0.001$ vs. KGN group, n = 8.

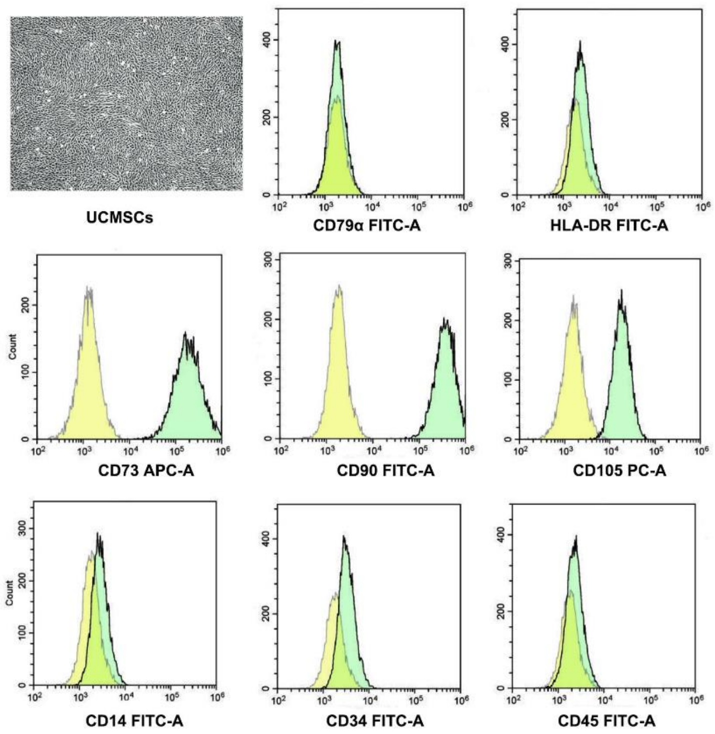

Figure S5 Identification of human umbilical cord mesenchymal stem cells (UC-MSC) by detecting the positive CD73, CD90 and CD105, and negative CD79α, HLA-DR, CD14, CD34 and CD45 mearkers by flow cytometry.

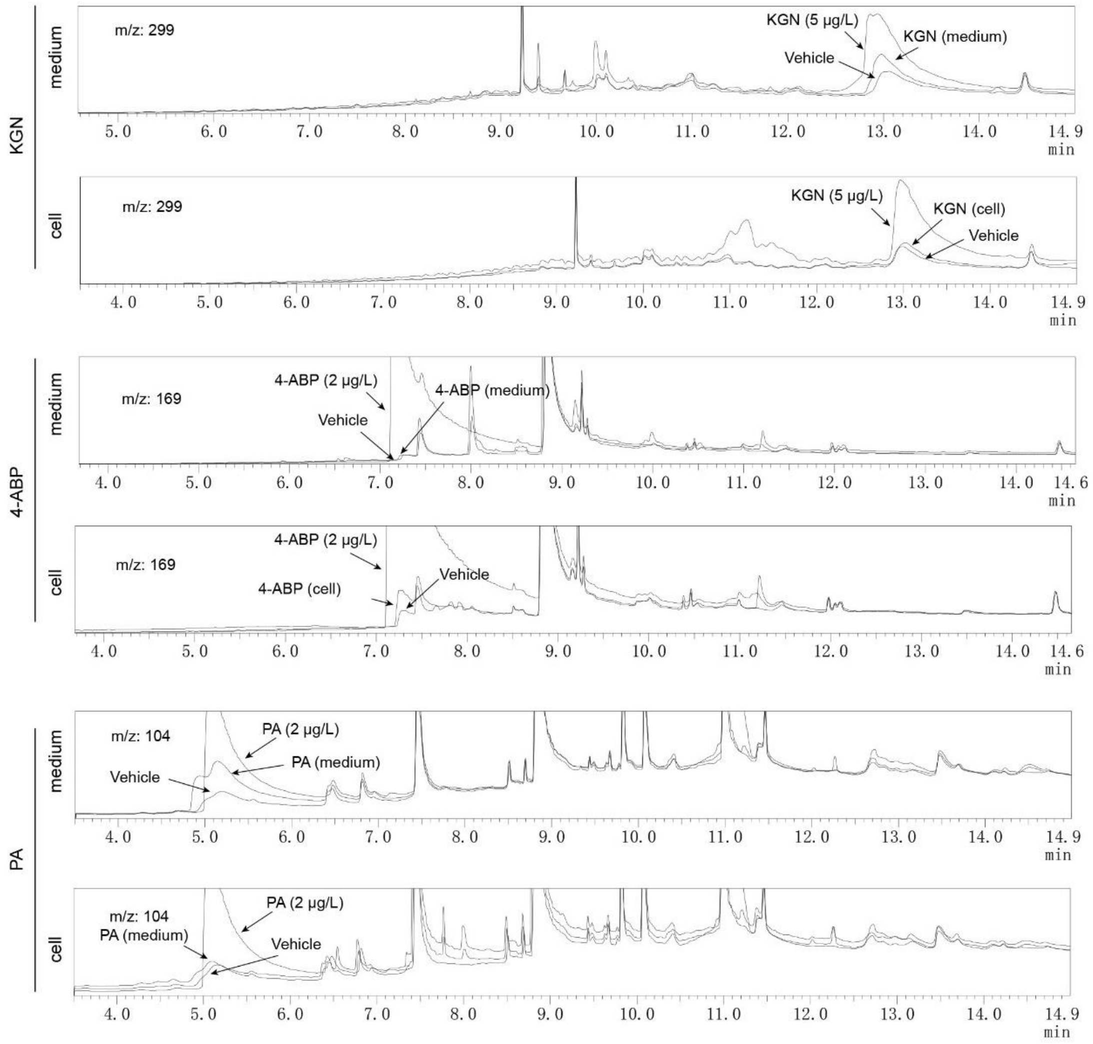

Figure S6 KGN, 4-ABP and PA detection by HPLC/MS in umbilical cord mesenchymal stem-cell (UC-MSC) culture medium and in UC-MSC 24 h after KGN exposure.

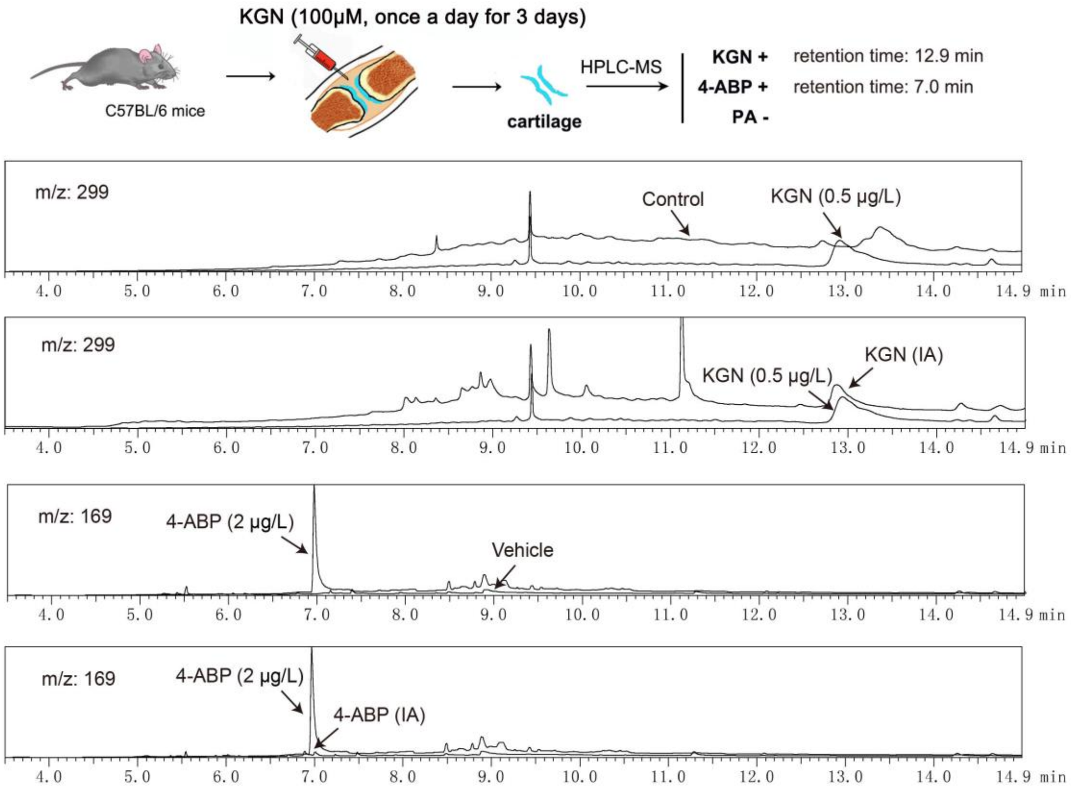

Figure S7 KGN and 4-ABP detection by HPLC/MS in cartilage after intra-articular KGN injection.

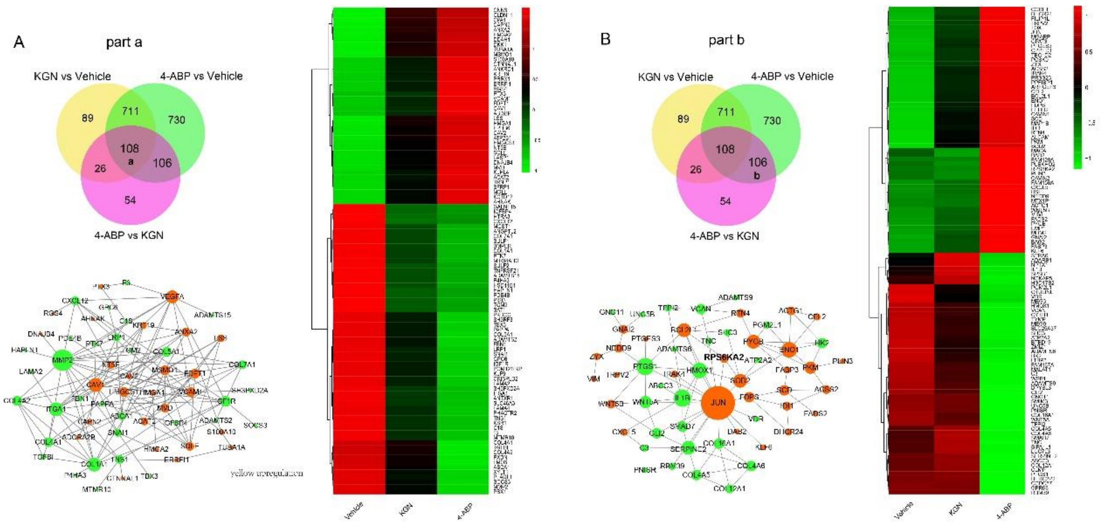

Figure S8 Bioinformatic analysis of umbilical cord mesenchymal stem-cell transcriptomic profile after treatment with KGN or 4-ABP (10 μM) for 3 days. The differentially expressed RNAs in part a (A) and b (B) of Venn diagram were used to construct the networks.

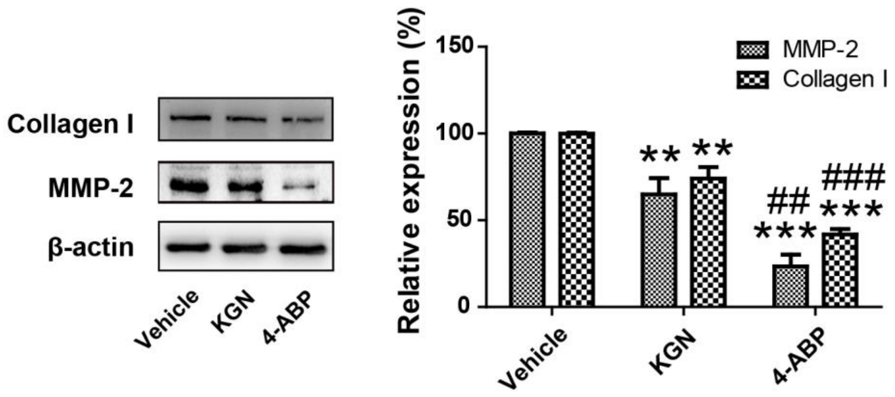

Figure S9 Collagen I and MMP-2 protien expression in umbilical cord mesenchymal stem-cells treated with 4-ABP or KGN for 4 days. **$p < 0.01$, ***$p < 0.001$ versus vehicle; $^{##}p < 0.01$, $^{###}p < 0.001$ versus KGN. n = 3 per group.
